# Compensatory growth as a response to post-drought in grassland

**DOI:** 10.3389/fpls.2022.1004553

**Published:** 2022-12-01

**Authors:** Huailin Zhou, Lulu Hou, Xiaomin Lv, Guang Yang, Yuhui Wang, Xu Wang

**Affiliations:** ^1^ State Key Laboratory of Severe Weather, Chinese Academy of Meteorological Sciences, China Meteorological Administration, Beijing, China; ^2^ Institute of Agricultural Resources and Regional Planning, Chinese Academy of Agricultural Sciences, Beijing, China; ^3^ College of Teacher Education, Capital Normal University, Beijing, China; ^4^ State Key Laboratory of Vegetation and Environmental Change, Institute of Botany, Chinese Academy of Sciences, Beijing, China

**Keywords:** compensatory growth, grassland ecosystem, drought, resilience, recovery, mechanism

## Abstract

Grasslands are structurally and functionally controlled by water availability. Ongoing global change is threatening the sustainability of grassland ecosystems through chronic alterations in climate patterns and resource availability, as well as by the increasing frequency and intensity of anthropogenic perturbations. Compared with many studies on how grassland ecosystems respond during drought, there are far fewer studies focused on grassland dynamics after drought. Compensatory growth, as the ability of plants to offset the adverse effects of environmental or anthropogenic perturbations, is a common phenomenon in grassland. However, compensatory growth induced by drought and its underlying mechanism across grasslands remains not clear. In this review, we provide examples of analogous compensatory growth from different grassland types across drought characteristics (intensity, timing, and duration) and explain the effect of resource availability on compensatory growth and their underlying mechanisms. Based on our review of the literature, a hypothetic framework for integrating plant, root, and microbial responses is also proposed to increase our understanding of compensatory growth after drought. This research will advance our understanding of the mechanisms of grassland ecosystem functioning in response to climate change.

## Introduction

Grasslands, as one of the world’s most widespread vegetation types, cover approximately 30% of the Earth’s land surface (Parton et al., 2012) and 69% of agricultural land area (Dixon et al., 2014), respectively. Grasslands not only serve as an important global reservoir of food production (Schirpke et al., 2019), but also play a critical role in the global carbon and water cycle, as well as plant-soil feedback to climate change (Bowman et al., 2015; Putten et al., 2016; Pugnaire et al., 2019). Grassland growth and productivity are largely regulated by temperature and soil water content, particularly the amount and timing of precipitation events (Knapp and Smith, 2001; Knapp et al., 2002; Huxman et al., 2004; Hufkens et al., 2016). In recent decades, ongoing global changes in temperature and precipitation have significantly increased the frequency, severity, and duration of drought events (Dai, 2012; IPCC, 2013; Trenberth et al., 2014; Cook et al., 2015), which also projected to continue to increase in the near future (Vicente-Serrano et al., 2020). The alterations in water availability before or during the growing season are weakening the stability and functionality of grassland ecosystems around the world, particularly in arid and semi-arid regions (Macdougall et al., 2013; Song and Yu, 2015). Because drought events could directly or indirectly affect plant community structure (Knapp et al., 2008; Cherwin and Knapp, 2012; Carlyle et al., 2014; Tielborger et al., 2014), threaten grassland productivity (Knapp and Smith, 2001; Volaire et al., 2014; Frank et al., 2015) and even cause grassland degradation (Breshears et al., 2005), and then alter carbon and nitrogen dynamics (Mackie et al., 2019). Naturally, to deal with the negative impacts of drought on grasslands functions and services, it is urgent to understand how grasslands respond to drought.

Many studies on grassland responses during drought have been well synthesized in both reviews (Grman et al., 2010; Niu et al., 2014; Hoover et al., 2018) and meta-analyses (Matos et al., 2019; Deng et al., 2021), which have considerably improved our understandings of the impacts of drought on grassland biotic and abiotic processes. For example, the mean effect of drought on aboveground net primary production (ANPP) is demonstrated to be negative (Hoover et al., 2014; Niboyet et al., 2017; Li et al., 2022). Droughts have legacy effects on bacterial and fungal community composition, which could, in turn, influence plant growth and ecosystem through plant-soil feedback (De Vries et al., 2012b; Kaisermann et al., 2017; Griffin-Nolan et al., 2018). Except for the ability of grasslands to resist drought (e.g., resistance), the recovery ability of grasslands after drought (e.g., resilience) is another important entry point for clarifying the responses of grasslands to drought (Vogel et al., 2012; Mori et al., 2013; Hoover et al., 2014; Oliver et al., 2015; Xu et al., 2021). However, the recovery ability of grasslands to different drought characteristics (e.g., timing, intensity, and duration) and climate contexts were rarely studied (Vilonen et al., 2022). Besides, our knowledge of grassland drought response is incomplete without understanding the responses after drought and to what extent grasslands can recover. Therefore, understanding the change patterns in the structure and function of grassland ecosystems during the period of after drought and exploring their underlying mechanisms, are crucial for forecasting grassland ecosystem function and dynamics under climate change.

**Figure 1:**
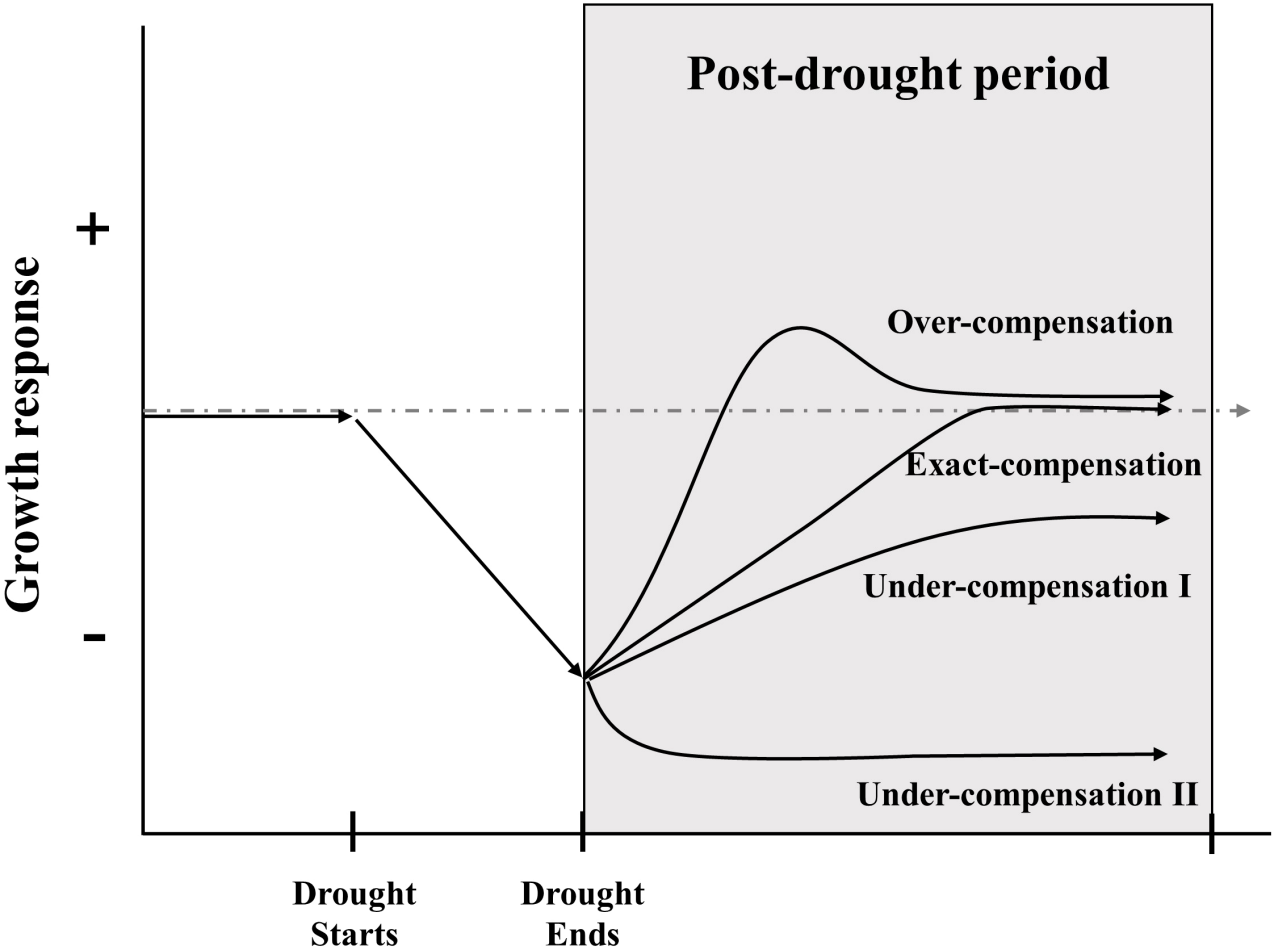
Framework for describing the growth response of grassland ecosystem after drought ends. The grey and dashed line represents growth response in condition without drought, the solid line indicated possible compensatory patterns under drought. Over-compensation means the growth rate increase rapidly after the end of a drought, then exceed the response level of control treatment to some extent, and finally reach the same response level of control treatment; exact-compensation represents the growth rate return to be comparable with control treatment after drought; under-compensation I indicates that the growth rate cannot reach the same level of control treatment with slow growth rate; under-compensation II denotes the collapse of grassland ecosystem with much slower growth rate (Frank et al., 2015; Li et al., 2021; Vilonen et al., 2022).

Compensatory growth (CG), defined as the accelerated growth response of plants to damage (Belsky, 1986), which is implied the ability of plants to offset the adverse effects of tissue damage, restore organic functionality, and maintain their original growth state after perturbations (Mcnaughton, 1983). CG has received wide acceptance as a survival strategy of organisms under stressful conditions and a fundamental mechanism for ecosystem stability (Mangel and Munch, 2005; Gonzalez and Loreau, 2009). In fact, it sometimes takes different names, like resilience, recovery, and compensatory dynamic; they all share the essential meaning that accelerated growth organism when recovering from a period of unfavorable conditions (Li et al., 2021). According to the relative strength of growth rate after disturbance compared to the undisturbed group, CG can be classified into three types: under-compensation, exact-compensation, and over-compensation (**Figure 1**) (Belsky, 1986; Li et al., 2021). Although the existence of CG is widely acknowledged in ecological systems but has received little attention in stress-ecological studies (Metcalfe and Monaghan, 2001; Gessler et al., 2020). In general, it is conventionally to constrain the period of CG assessment through a pre-defined post-drought period or to the status where growth returns to a historic norm (Ovenden et al., 2021). The capacities of grassland CG are different among species (Knapp et al., 2015), community components (Carlsson et al., 2017; Stampfli et al., 2018; Wilcox et al., 2020), life forms (Volaire, 2003; Nippert and Knapp, 2007), nutrient stress tolerances (Macgillivray et al., 1995; Bharath et al., 2020), interactions among soil microbes (De Vries et al., 2012a; Fry et al., 2018) and disturbance’s properties (Chen et al., 2020; Saeidnia et al., 2020; Li et al., 2022). Besides, CG could be evaluated by a variety of quantitative indicators, such as productivity, biomass, species number, coverage, and so on (Yuan et al., 2020). Recent research on the prevalence and detection of CG leaves a large gap in the knowledge of the mechanisms that affect the temporal and scale of CG (Kahl et al., 2019; Li et al., 2020; Saeidnia et al., 2020; Hossain and Li, 2021; Jiao et al., 2021; Li et al., 2021; Ovenden et al., 2021; Vilonen et al., 2022). Thus, a deeper understanding of the pattern of grassland CG and its variation is a major challenge for the emerging extreme climate events and human disturbance.

Here we reviewed current knowledge on the CG of grasslands to drought stress. We firstly discuss differences in CG response to drought imposed by manipulated experiments or natural precipitation variations. Then, CG patterns under different drought timing, intensity, and duration were compared and discussed. Meanwhile, as plant-soil feedback plays a key role in the CG response to drought, resource availability was also addressed in the text. In the following section, we concluded the underlying mechanisms of CG in response to drought across biotic and abiotic reasons. Finally, suggestions for future research were also given to deepen our understanding of the responses during the period after drought and benefit for forecasting grassland ecosystem function and dynamics under climate change.

## Compensatory growth among different grassland ecosystems

Numerous rainfall manipulation experiments have been conducted to investigate the growth responses of different grassland ecosystems after drought (Hoover et al., 2018; Matos et al., 2019). In the mesic grasslands of North America and Switzerland, ANPP can fully recover (the same as exact-compensation) within a single year after a short-term extreme drought with grass species compensating for the decreased forb productivity (Hoover et al., 2014; Stampfli et al., 2018; Mackie et al., 2019; Wilcox et al., 2020). Besides, a study focused on belowground net primary productivity (BNPP) also suggested that drought-induced reductions in root production can recover rapidly in a coming wet year even though the drought legacy effects may persist for years after drought (Slette et al., 2022). By contrast, the CG of burned sites was mostly contributed to annual forb ANPP compensating for reduced grass ANPP, while the CG of unburned sites was promoted by subdominant annual and perennial grass species in a savanna grassland in South Africa (Wilcox et al., 2020). The opposite roles played by forbs and grass species mentioned above might be mainly due to the difference in community composition in these sites. As forbs are often less resistant to drought than grasses, if the plant community was dominated by forbs, then the CG of the total ANPP may decrease (Xu et al., 2021). Additionally, annual forbs are more resilient than perennial forbs, which are characterized by limited seedling recruitment and slow regrowth from surviving belowground organs after drought (Wilcox et al., 2020; Xu et al., 2021).

Meanwhile, semi-arid grassland seems to require more time for the ANPP to achieve exact-compensation from drought. Xu et al. (2021) explored the recovery potential of ANPP by inducing two years of extreme drought (66% reduction in ambient growing season precipitation) followed by two years of recovery (ambient precipitation) in a semi-arid grassland ecosystem in Inner Mongolia, China. The results show that ANPP decreased by approximately 33% during the two years of extreme drought. However, one year after the extreme droughts, the ANPP of the drought plots returned to 83% of the ambient plots and fully recovered to ambient ANPP by the second year. The authors attributed these differences to three points: (a) the lower precipitation efficiently limits CG in the semi-arid regions; (b) the reduction of ANPP in the semiarid grassland is much higher than that in the mesic grassland (Ma et al., 2020), which increase the recovery time; (c) a large proportion of high resistance and low resilience of perennial forb species may delay the recovery time of the semiarid grassland (Tello-García et al., 2020).

As for arid grasslands, the CG rate may more slowly due to greater resource limitations and more severe impacts (Stuart-Haentjens et al., 2018). A study in Inner Mongolia suggested that the net primary productivity was less affected by light to moderate drought than moderate to severe drought (Liu et al., 2021). At the same time, another study conducted in the Chihuahuan Desert found that drought consistently and strongly decreased the cover of a dominant C4 grass (*Bouteloua eriopoda*), whereas water addition slightly increased the cover, even with little variation between years (Báez et al., 2013). The limited CG of *Bouteloua eriopoda* responding to increased water availability may reflect morphological constraints on this rhizomatous grass (Báez et al., 2013).

In general, grasslands are composed of two dominant herbaceous functional groups: grass and forb, which show great differences in their vulnerability to extreme drought (Taylor et al., 2011; Wilcox et al., 2020). Grass species are generally better able to tolerate drought, especially C4 grasses, whereas forb species may avoid drought *via* deeper rooting profiles (Nippert and Knapp, 2007). Besides, the growth responses of annual and perennial species may be different during and after drought (Volaire, 2003). Therefore, CG in grasslands may depend on function diversity in predrought communities (Stampfli et al., 2018; Wilcox et al., 2020). However, a study conducted in 13 extreme natural-drought experiments spreading over two biogeographic regions (five sites in annual-dominated grasslands in California and eight sites in perennial-dominated grasslands in the Great Plains) suggested there was no correlation between pre-drought plant diversity and post-drought resilience (Bharath et al., 2020). More importantly, the productivity of grassland ecosystems is simultaneously co-limited by nutrients and water across a wide range of precipitation (Bharath et al., 2020). For example, species-rich semi-natural grasslands exhibited a lower CG compared with intensively managed agricultural grasslands (De Keersmaecker et al., 2016).

## Compensatory growth response to drought intensity, timing, and duration

Drought intensity, timing, and duration are fundamental characteristics of experimental or natural drought events. Drought stress can cause a series of reductions in morphological and physiological functional traits (e.g., plant height, specific leaf area, length of roots, leaf water potential, and photosynthetic capacity), which may finally lead to a reduction in productivity (Cenzano et al., 2013; Wellstein et al., 2017). Response diversity, describing the variation of responses to environmental change among species in a particular community, maybe a key determinant of ecosystem stability and functionality (Elmqvist et al., 2003; Mori et al., 2013). For instance, perennial caespitose grasses and rhizomatous grasses showed different growth response strategies to drought, as the CG of rhizomatous grasses declined with increasing water stress intensity while caespitose grasses displayed little CG with strong drought resistance (Zhang et al., 2018). Besides, defoliation could stimulate the CG of rhizomatous grasses under wet conditions, but the positive effects of defoliation can be weakened by drought intensity (Zhang et al., 2018). Even though, the CG of grassland dominated by perennial species almost remains constant with increasing drought intensity (Ruppert et al., 2015). The main reason may be contributed to the degree of drought intensity being below the upper limit that could cause ecosystem collapse (Dechant and Moradkhani, 2015).

The responses of grassland ecosystems to drought may vary with different seasonal drought timing. When droughts occur in the early season, the reductions in current-year biomass appear to be large enough due to the limitation length of a peak growth period for biomass accumulation (Meng et al., 2019). On contrary, when droughts happen in the late season, the decreased biomass will be reflected in the following year because of large negative legacy effects (Jiao et al., 2021). For example, the timing of drought significantly decreased ANPP (18%~26% reduction compared to the control treatments) during the growing season in a mesic grassland, with later droughts (early summer drought and late summer drought, respectively) having a larger effect than earlier drought (late spring drought), while BNPP was not significantly affected by any manipulated drought timing (Denton et al., 2016). Similar findings were also confirmed in a meadow steppe where spring and summer droughts decreased ANPP but did not affect BNPP (Meng et al., 2019). Furthermore, grasslands subjected to mid-summer drought tend to be primed for greater CG in the following year than grasslands experiencing earlier drought in the season (De Vries et al., 2012b; Denton et al., 2016).

The duration of drought is important to explain the variability of CG, with longer droughts resulting in slower grassland CG, through causing the depletion of seedbank and stored resources needed for re-establishment and resprouting of the drought-sensitive species (Ruppert et al., 2015; Estiarte et al., 2016; Matos et al., 2019). In the North American semi-arid grassland biome, reductions in ANPP appeared to be greater when the rainfall patterns of the growing season were dominated by many small events (that is, chronic drought), while it turned out to be not when rainfall patterns were characterized by large rain events (Cherwin and Knapp, 2012). Compared with wood biomes, grasslands exhibited a stronger CG when exposed to chronic drought, by contrast, displayed a weaker CG when exposed to intense drought (Jiao et al., 2021). Furthermore, the adverse effects of intense drought on ANPP were found to be more significant than chronic drought, additionally, drought duration appeared to hardly alter this pattern (Carroll et al., 2021). Therefore, the compensation of grassland ANPP in response to future droughts may be reduced when the rainfall regimes of the growing season being more extreme.

## Compensatory growth under different resource availability

The amount of CG can be also affected by resource availability through the plant-soil feedback (Van Staalduinen et al., 2009). Due to changes in the soil water availability during and after drought, the turnover of C and N in soils is also altered. Increased duration and intensity of drought are usually associated with decreasing C and N mineralization and inorganic N fluxes (Borken and Matzner, 2009; Deng et al., 2021). Even though, a pulse in net C and N mineralization following the wetting of dry soil is generally observed (Wu and Brookes, 2005). Previous grassland studies indicate that drought stresses alleviate N limitation and have a positive effect on forage quality (Dumont et al., 2015). Additionally, increasing N deposition resulting from anthropogenic N emissions can improve grassland CG after a drought even in arid environments (Kinugasa et al., 2012). Besides, the wetting pulses have a greater impact on C and N mineralization or flux rates in arid and semiarid grasslands than that in humid and subhumid grassland (Borken and Matzner, 2009). Whereas, it is worth noting that the cumulative C and N mineralization are most likely less compared with soil under optimum moisture even with wetting pulses, which implies that wetting pulse cannot compensate for small mineralization rates during periods (Borken and Matzner, 2009). In another word, the grassland CG pulsed by the short-term C and N mineralization may be not sustainable if the droughts become frequent.

The dynamics of soil C and N are affected not only by plant organic matter input but also by microbial activities (Deng et al., 2021). Soil microorganisms participate in all aspects of C and N dynamics, regulate the formation of soil organic matter and release extracellular enzymes through C and N turnover (Ren et al., 2017). Meanwhile, microbial decomposition of soil organic matter can cause CO_2_ efflux and gaseous N emissions by producing C and N-degrading extracellular enzymes (Ren et al., 2017). Interestingly, both microbial and extracellular enzyme activities appear to be more sensitive to soil water content and temperature than to their nutritional resources (Nielsen and Ball, 2015). Therefore, drought can alter soil microbial composition and enzyme activities, then affect the soil C and N balance (Ren et al., 2017), and finally influence both the belowground and aboveground performances of plants in response to drought.

However, previous studies have no consistent results on the relationship between CG and resource availability. Some studies suggested that plants tend to overcompensate more frequently under unfavorable growth conditions (Coughenour et al., 1990; Hawkes and Sullivan, 2001). In contrast, some researcher insisted that CG only occur under abundant even optimal conditions (Belsky et al., 1993), known as the compensatory continuum hypothesis (CCH). Furthermore, even for the same functional type, CG varies among different resource levels (Hawkes and Sullivan, 2001). For instance, *Leymus chinensis* had less CG under dry conditions compared with wet conditions, while it is opposite for *Stipa krylovii* (Van Staalduinen and Anten, 2005). Besides, the chronic nutrient addition in the Great Plains reduced grassland drought resistance and increased drought resilience regardless of annual-dominated or perennial-dominated grassland (Bharath et al., 2020). Based on CCH, a limited resource model (LRM) was once introduced to explain the range of observed effects of resource levels on and prediction for compensation for herbivory (Wise and Abrahamson, 2005), which suggested that CG depends on the type of resource and disturbance intensity (e.g., drought, heat stress, herbivore) under consideration (Metcalfe and Monaghan, 2001; Michael and Warren, 2007). The LRM model introduced the roles of limiting and non-limiting resources, and analyzed which resource was affected by the disturbance. If the disturbance mainly affects the first limiting resource, then higher CG is expected to occur at high resource availability. The application of the LRM model needs to take certain experimental requirements into account, including full factorial experimental design, consistent levels of disturbance across environments, determining whether a focal resource is liming plant fitness, and identifying the resource affected by disturbance (Wise and Abrahamson, 2005). Even though, the application of LRM to predict CG in response to drought still remain with many uncertainties and need to be tested in future study, with the complex backgrounds of climate region, grassland type, and species diversity (Monaghan, 2008).

## Underlying mechanisms of compensatory growth in response to drought

**Figure 2:**
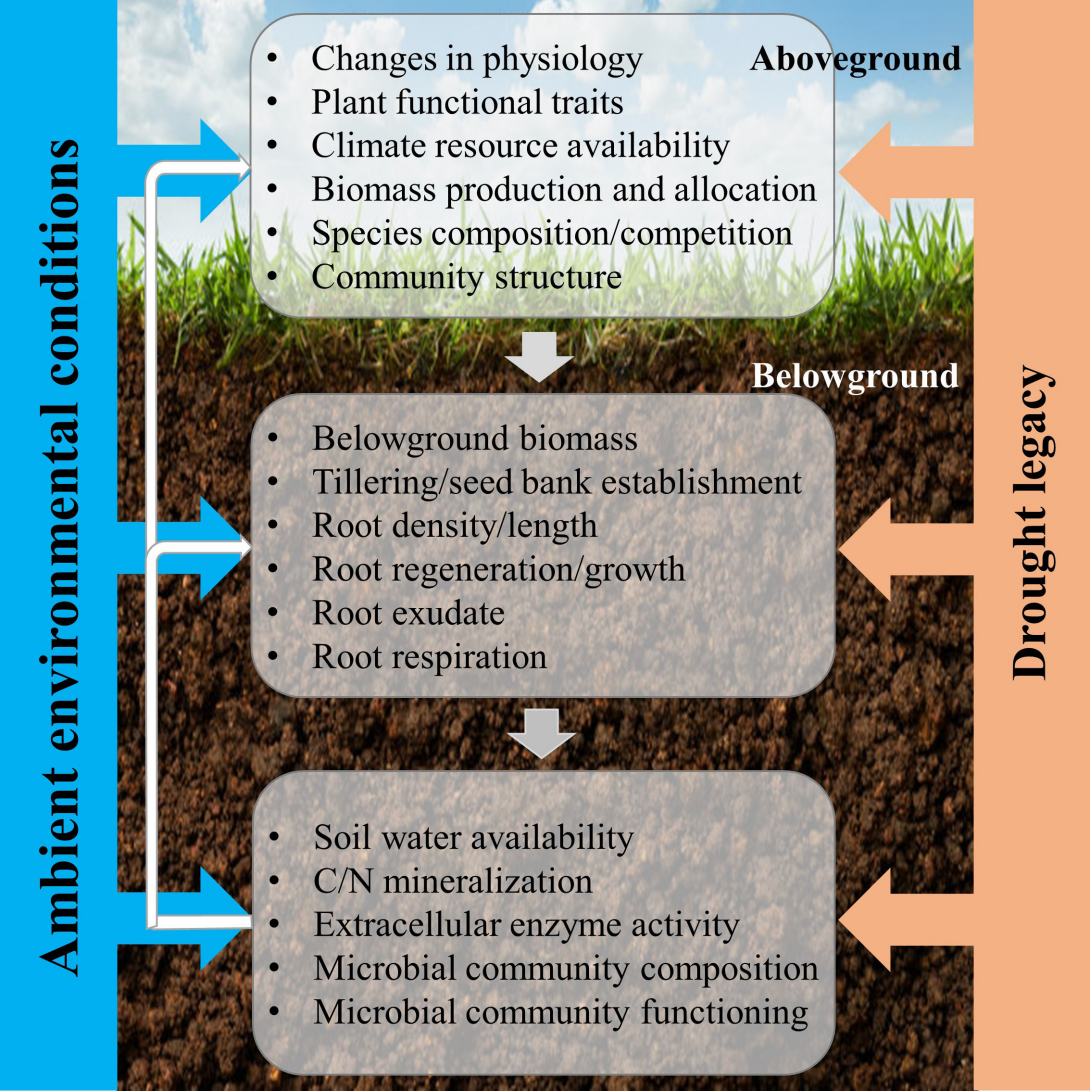
Conceptual framework of the responses of aboveground and belowground processes to post-drought for compensatory growth. The three main three components include aboveground plant, root, and soil microorganism, which are determined by plant community trait, soil type and climate (ambient environmental conditions). The effects of drought on the three components are achieved individually, but we hypothesize that the responses of the aboveground plant determine the responses of root after drought, thereby, the microbial community responses, which react on aboveground plant growth, community composition, ultimately, ecosystem stability and function (Williams and De Vries, 2020).

The CG response of grassland to drought is a synthesis result of plant, root, and soil feedback (**Figure 2**). With respect to aboveground plant responses, CG under drought conditions may be triggered by the recovery of existing individuals within a grassland ecosystem, or by compensatory dynamics where particular individuals or species increase in abundance to counterbalance reductions in other individuals or species (Tilman and Downing, 1994). Compensatory effects, arising from various responses of different plants or functional groups to perturbations, are an important mechanism for sustaining ecosystem stability (Gonzalez and Loreau, 2009; Song and Yu, 2015). Besides, compensatory changes in species population in response to environmental fluctuations can maintain an appreciated steady state between the rate of resource supply and its consumption (Morgan Ernest and Brown, 2001). The difference in performance between functional traits is a good indicator of plant growth strategy in response to post-drought. For example, *Leymus chinensis* exhibited a greater capacity for CG than *Stipa krylovii*, because it has a stronger ability in storing carbohydrates and reallocating them after leaf losses, and a more positive effect of defoliation on light penetration through the canopy (Van Staalduinen and Anten, 2005). The CG of the different grassland ecosystems responding to drought may occur through two fundamentally different biotic mechanisms (Wilcox et al., 2020): (1) drought-tolerant plants increase in abundance and functionally compensate for declines in drought-intolerant species, which is called compensatory dynamics (Gonzalez and Loreau, 2009; Hoover et al., 2014); (2) all individuals within the community recover fully after drought, which is defined as physiological compensatory (Connell and Ghedini, 2015). Physiological compensation often happens with short-term and (or) moderate drought, while compensatory dynamics are more likely to occur under long-term and (or) extreme drought by rearranging species abundances (Smith, 2011).

Nevertheless, plant roots and microbial community also play a key role in mediating the post-drought responses (**Figure 2**). Root traits, like specific root length, root dry matter content, and toot tissue density, are important for shaping post-drought responses and vary across a range of grassland species with different growth strategies (De Vries et al., 2016; Williams and De Vries, 2020). In addition, root exudates form a pathway for plant-microbial communication and have the potential to influence plant tolerance and recovery during and/or after drought (Williams and De Vries, 2020). The drought-induced changes in the quality of root exudates might have implications for the recovery of plants and microbes (De Vries et al., 2019). Besides, the structure of the microbial community can determine the functional responses of the grassland ecosystems, through the expression of functional genes. Furthermore, CG was almost certainly promoted by a drought-induced increase in soil N availability as a higher mineral N supply rate appeared in the month after rewetting, and then increased plant nitrogen content two months after rewetting (Mackie et al., 2019). Due to an increase in plant-available N, the plant photosynthetic activities are upregulated during post-drought, and then drive a short-term increase in forage quality (Bloor and Bardgett, 2012; Niboyet et al., 2017). Therefore, this field of research will need to be driven forward by studying general mechanisms, focusing on mechanisms that link below- and aboveground processes and responses (**Figure 2**).

## Limitations and suggestions for future research

With growing concerns about grassland vulerability, a comprehensive understanding of grassland response to drought is becoming increasingly important. Previous studies have mainly focused on the plant or ecosystem responses during drought, however, there is still limited understanding of the period after drought. Here, we reviewed compensatory growth across grassland types, drought characteristics, and resource availabilities. Besides, the underlying mechanisms of CG were also summed up. However, there is still a lot of work to achieve the ultimate target on how to accurately quantify CG and predict its direction and strength under changing ambient environmental conditions. The followings are some suggestions for further research on CG.

Firstly, the assessment method of CG mentioned above implicitly assumes that the reference growth level (the pre-defined post-drought period or the historic norm status) is where the drought legacy ends. However, the legacy of drought might be extending far beyond a return to reference growth level under some conditions (Ingrisch and Bahn, 2018; Ovenden et al., 2021). Thus, when CG is activated and then how long it will take are still debatable, which need to be finely defined in future study. Besides, as plant functional traits play an important role in determining net carbon assimilation and allocation, therefore, a better understanding of the post-drought recovery performance of plant functional traits could improve our ability to predict grassland ecosystem production in a rapidly changing climate (Yin and Bauerle, 2017). In order to improve the evaluation accuracy of CG, filtering out suitable plant-soil functional traits may be a good pathway (**Figure 2**).

Secondly, the adverse impacts of a single drought might be reflected in plant water and nutrient acquisition than in ecosystem carbon cycling, while both sides could be emphasized by a second drought or repeated droughts (Slette et al., 2022). The potential consequences of repeated drought on CG may range from increased adaptation to increased sensitivity, which remains unclear (Slette et al., 2022). Some studies have suggested that the adaptation of soil microbial communities to a previous drought can increase the drought tolerance of plants in facing a subsequent drought event (Lau and Lennon, 2012; Meisner et al., 2013). Besides, drought-exposure history could increase complementarity between plant species in response to future droughts (Chen et al., 2022). Due to more frequent droughts are expected in many parts of the world in the future, studies on the CG response to repeated droughts are needed to improve our knowledge of grassland stability.

Thirdly, even though extensive studies have been focusing on the effects of drought on grasslands, there is limited understanding of the period after drought due to a lack of studies on belowground responses and an undue emphasis on aboveground ecosystem responses (Vilonen et al., 2022). ANPP is the most common indicator or function for evaluating the aboveground ecosystem responses to drought (Hoover et al., 2014; Knapp et al., 2015), because aboveground biomass is easier to obtain than belowground biomass. In fact, grasslands allocate a substantial portion of total net primary production to roots and then store most of their carbon belowground (Hui and Jackson, 2006; Silver et al., 2010). Belowground responses, such as BNPP and soil CO_2_ flux, are of particular importance in determining the size of the soil carbon pool (Post et al., 1982; Slette et al., 2021). Previous studies have demonstrated that the CG of ANPP and BNPP is different over time, like average precipitation amounts are sufficient for CG in ANPP after extreme drought, while CG in BNPP might be more resource-demanding (Slette et al., 2022). Due to the different patterns of ANPP and BNPP response to changes in water availability, belowground processes and their underlying mechanisms should be addressed in future work modeling ecosystem responses to climate change (Denton et al., 2016).

## Conclusion

Overall, in light of the persistence and intensification of climate change, the responses of ecosystems to drought need to be paid more attention. In past decades, a series of analogous compensatory growth of net primary productivity and community stability to drought disturbance in different grassland ecosystems were discussed based on effective indicators, like recovery and resilience. In this review, we discussed compensatory growth across different grassland ecosystems and drought characteristics, explained the effect of resource availability on compensatory growth, and summed up the mechanism by which compensatory growth may occur. The review suggests that the CG is likely to be primarily due to the different responses of plant functional groups and their interactions with soil microbes to water availability. We propose identifying the starting time and duration of compensatory growth; better describing the symbol of CG with plant-soil functional traits; conducting more research on the plant-soil feedback and the decoupling of above- and belowground processes. These proposed researches would expand our understanding of compensatory growth, and increase our ability to evaluate the stability and sustainability of grassland ecosystems in the face of climate change.

## Author contributions

HZ, LH, XL, GY, YW and XW jointly conceived and wrote the paper. All authors contributed to the article and approved the submitted version.

## Funding

This work was financially supported by the Second Tibetan Plateau Scientific Expedition and Research (STEP) program (2019QZKK0106) and the National Natural Science Foundation of China (42205126, 41975145, 41775108).

## Acknowledgments

The authors thank the reviewers for the constructive comments on an earlier version of this manuscript.

## Conflict of interest

The authors declare that the research was conducted in the absence of any commercial or financial relationships that could be construed as a potential conflict of interest.

## Publisher’s note

All claims expressed in this article are solely those of the authors and do not necessarily represent those of their affiliated organizations, or those of the publisher, the editors and the reviewers. Any product that may be evaluated in this article, or claim that may be made by its manufacturer, is not guaranteed or endorsed by the publisher.
